# PIP5KIβ Selectively Modulates Apical Endocytosis in Polarized Renal Epithelial Cells

**DOI:** 10.1371/journal.pone.0053790

**Published:** 2013-01-16

**Authors:** Christina M. Szalinski, Christopher J. Guerriero, Wily G. Ruiz, Brianne E. Docter, Youssef Rbaibi, Núria M. Pastor-Soler, Gerard Apodaca, Manojkumar A. Puthenveedu, Ora A. Weisz

**Affiliations:** 1 Renal Electrolyte Division, University of Pittsburgh Medical School, Pittsburgh, Pennsylvania, United States of America; 2 Department of Cell Biology, University of Pittsburgh Medical School, Pittsburgh, Pennsylvania, United States of America; 3 Department of Biological Sciences, University of Pittsburgh, Pittsburgh, Pennsylvania, United States of America; 4 Grand Valley State University, Allendale, Michigan, United States of America; 5 Department of Biological Sciences, Carnegie Mellon University, Pittsburgh, Pennsylvania, United States of America; University of Birmingham, United Kingdom

## Abstract

Localized synthesis of phosphatidylinositol 4,5-bisphosphate [PtdIns(4,5)P_2_] at clathrin coated pits (CCPs) is crucial for the recruitment of adaptors and other components of the internalization machinery, as well as for regulating actin dynamics during endocytosis. PtdIns(4,5)P_2_ is synthesized from phosphatidylinositol 4-phosphate by any of three phosphatidylinositol 5-kinase type I (PIP5KI) isoforms (α, β or γ). PIP5KIβ localizes almost exclusively to the apical surface in polarized mouse cortical collecting duct cells, whereas the other isoforms have a less polarized membrane distribution. We therefore investigated the role of PIP5KI isoforms in endocytosis at the apical and basolateral domains. Endocytosis at the apical surface is known to occur more slowly than at the basolateral surface. Apical endocytosis was selectively stimulated by overexpression of PIP5KIβ whereas the other isoforms had no effect on either apical or basolateral internalization. We found no difference in the affinity for PtdIns(4,5)P_2_-containing liposomes of the PtdIns(4,5)P_2_ binding domains of epsin and Dab2, consistent with a generic effect of elevated PtdIns(4,5)P_2_ on apical endocytosis. Additionally, using apical total internal reflection fluorescence imaging and electron microscopy we found that cells overexpressing PIP5KIβ have fewer apical CCPs but more internalized coated structures than control cells, consistent with enhanced maturation of apical CCPs. Together, our results suggest that synthesis of PtdIns(4,5)P_2_ mediated by PIP5KIβ is rate limiting for apical but not basolateral endocytosis in polarized kidney cells. PtdIns(4,5)P_2_ may be required to overcome specific structural constraints that limit the efficiency of apical endocytosis.

## Introduction

Clathrin-mediated endocytosis is a multi-step process of cargo internalization from the plasma membrane that is essential for the regulation of cell receptor density and uptake of nutrients essential for cell function [1]. For example, cholesterol ingested in the diet is packaged into lipoprotein particles that are internalized via ubiquitously expressed cellular LDL-receptors to enable distribution of the lipid to peripheral tissues [2]. Similarly, the transferrin receptor mediates internalization of iron loaded transferrin from the cell surface to maintain iron homeostasis. The incorporation of these and other diverse cargoes into forming clathrin-coated pits (CCPs) is facilitated by endocytic adaptors proteins, including AP-2, epsin, autosomal recessive hypercholesterolemia (ARH), and disabled 2 (Dab2) [3]. In addition to recruiting cargo, these proteins also recruit additional factors necessary for membrane invagination [3], [4]. In turn, the lipid phosphatidylinositol 4,5-bisphosphate [PtdIns(4,5)P_2_] plays a key role in recruitment of clathrin adaptors and other regulatory proteins critical for endocytosis to the plasma membrane [5].

The majority of cellular PtdIns(4,5)P_2_ is synthesized by phosphorylation of phosphatidylinositol 4-phosphate at the D-5 position of the inositol ring by phosphatidylinositol 4-phosphate 5-kinases type I (PIP5KI). Three isoforms of this enzyme exist (PIP5KIα, β, and γ) that are widely expressed in mammalian tissues [6], [7], [8], [9], [10]. Each of these isoforms of PIP5KI has been shown to be involved in clathrin mediated endocytosis in distinct cell types [11], [12], [13], [14].

PtdIns(4,5)P_2_ is localized to both the apical and basolateral plasma membrane domains of polarized epithelial cells [15], [16], [17]. The coordinated synthesis and degradation of PtdIns(4,5)P_2_ is necessary for execution of four critical steps in clathrin-mediated endocytosis [5], [18], [19], [20], [21]. First, PtdIns(4,5)P_2_ recruits clathrin adaptors that bind to PtdIns(4,5)P_2_ via distinct structural domains [22], [23], [24], [25]. After clathrin adaptors recruit clathrin and a coated pit is formed, dynamin is recruited to the membrane by binding to PtdIns(4,5)P_2_, where it then promotes scission of the vesicle [19]. The vesicle is then internalized by actin motor myosin VI, which also binds to PtdIns(4,5)P_2_ on the membrane [26]. Finally, after internalization, the hydrolysis of PtdIns(4,5)P_2_ by the 5′ phosphatase synaptojanin is important for vesicle uncoating [27].

Despite the critical role for PtdIns(4,5)P_2_ in endocytosis, it is unclear how changes in cell surface PtdIns(4,5)P_2_ levels affect endocytosis. Acute hydrolysis of PtdIns(4,5)P_2_ using targeted delivery of PtdIns(4,5)P_2_ phosphatase domains to the membrane causes rapid and profound blockade in endocytosis [28], [29], [30], however, the effects of less drastic manipulations on PtdIns(4,5)P_2_ levels has not been rigorously tested. Given the differences in PtdIns(4,5)P_2_ binding domains of adaptor proteins, one might speculate that changes in surface PtdIns(4,5)P_2_ levels might lead to selective recruitment of subpopulations of these proteins to CCPs resulting in selective internalization of certain cargoes. Indeed, CCPs enriched in individual cargoes have been described by several groups [3], [4], [31], [32].

Changes in PtdIns(4,5)P_2_ might modulate the efficiency of endocytosis at specialized plasma membrane domains. For example, endocytosis of the same cargoes from the apical surface of polarized cells proceeds considerably more slowly compared with internalization from the basolateral domain of the same cells or in non-polarized cells [33], [34], [35], [36], [37]. These differences may be due to steric constraints of the apical membrane, as CCP formation is limited to the base of microvilli, or to differences in cytoskeletal dynamics. Actin filaments are concentrated in the subapical terminal web and in microvilli, and may inhibit CCP invagination, fission, and/or vesicular transport. Actin appears to be critical for apical endocytosis as disruption of actin polymerization by cytocholasin D or jasplakinolide inhibits apical but not basolateral or non-polarized endocytosis [35], [38], [39], [40]. Actin as well as the actin nucleators Neural-Wiskott Aldrich Syndrome Protein and Arp2/3 are recruited to sites of clathrin endocytosis [41], [42]. The activities of these actin nucleators is enhanced by PtdIns(4,5)P_2_ [43], [44], [45] implicating PtdIns(4,5)P_2_ in actin dynamics involved in endocytosis.

In this study, we have examined the requirement for PtdIns(4,5)P_2_ in apical and basolateral endocytosis in polarized kidney epithelial cells by testing the effects of overexpressing PIP5KI isoforms on these pathways. Our work demonstrates that synthesis of PtdIns(4,5)P_2_ is rate limiting for apical but not basolateral endocytosis, and suggests a possible mechanism by which increased PtdIns(4,5)P_2_ levels enhance apical internalization efficiency. To our knowledge, this is the first study to compare the effects of PIP5KI overexpression on polarized endocytosis.

## Materials and Methods

### RT-PCR

Ambion RNAqueous phenol-free total RNA isolation kit was used to extract RNA. One µg of RNA, 2 µl of Oligo(dT)Primer (Ambion) and nuclease-free water in a total volume of 12 µl was mixed gently, heated for 3 min at 72°C, and placed immediately on ice. After centrifuging briefly, 2 µl of 10x RT buffer (Ambion), 2 µl 2.5 mM dNTP mix (Invitrogen), 0.5 µl of RNAse inhibitor (Ambion), 0.5 µl Moloney Murine Leukemia Virus Reverse Transcriptase (MMLV-RT; Ambion) enzyme (or water for control), and 2 µl nuclease-free water were added and the sample was incubated at 42°C for 1 h, followed by 92°C for 10 min to inactivate the RT enzyme. A 3 µl aliquot of this reaction was mixed with 2.5 µl of 10 µM sense and antisense primers, 5 µl of 10x PCR buffer, 0.5 µl of enzyme (GeneAmp High Fidelity), 5 µl of DMSO, and 26.5 µl of PCR grade water, placed into a 0.6 ml thin walled tube, and incubated in a Bio-Rad thermocycler. The cycle started at 95°C for 1 min and the following steps were then repeated 25 times: 95°C for 30 sec, 58°C for 30 sec, 72°C for 30 sec, followed by a single incubation at 72°C for 5 min and a hold at 4°C. Fifteen µl of the reaction mixture was electrophoresed on a 2% agarose gel. The primer sequences used were as follows: actin 5′-ACCTTCAACTCCATCATGAAG-3′ and 5′-CTGCTGGAAGGTGGACAG-3′, mouse PIP5KIα 5′-CACTGTCTCCCCTTCCTCTG-3′ and 5′-AGGAACAATGTCCAGCCAGT-3′, mouse PIP5KIβ 5′-AACTTCCCCCACTGCAGAAT-3′ and 5′-GTCTTCATGGTCAGCAAGCA-3′, mouse PIP5KIγ 5′-AAGGAGGAGGGTGCAGGAGT-3′ and 3′-GGGAGGGAGAACAAGGTT-3′.

### Generation of DNA Constructs and Adenovirus Production

Constructs encoding the glutathione S transferase (GST) tagged phosphotyrosine binding (PTB) domain of mouse Dab2 (residues 1–205) [46] and the GST tagged epsin N-terminal homology (ENTH) domain of rat epsin (residues 1–162) were provided by Dr. Pietro de Camilli (via Dr. Linton Traub) [47]. Rat Epsin-GFP was a gift from Dr. Pietro De Camilli (via Dr. Linton and the GFP tag was made monomeric by our lab by mutating position A206K. Epsin-GFP and was subcloned into the pAdtet vector. In this manuscript, we use the accepted convention of human nomenclature for murine PIP5KIβ and PIP5KIα, but note that our previous publications used the reverse mouse nomenclature [11], [34], [48]. Recombinant adenoviruses were generated from epsin-GFP and PIP5KI isoforms as described previously [48], [49], [50]. Generation of control non-expressing (M2-Rev), rabbit polymeric immunoglobulin receptor (pIgR), and transactivator adenoviruses was described previously [48], [49], [50]. Transactivator is required for expression from our doxycycline-repressible adenovirus constructs and was provided by co-infection of transactivator-expressing adenovirus or by using cells that stably express transactivator (see below).

### Cell Culture and Adenoviral Infection

mpkCCDc14 cells (mCCD cells) derived from the cortical collecting ducts of SV40 transformed mice [51] were provided by Dr. A. Vandewalle via Dr. John Johnson. Cells were cultured in Dulbecco’s modified Eagle’s medium (DMEM)/Ham’s F12 with 5 µg/mL insulin, 0.02 µg/mL dexamethasone, 0.01 µg/mL selenium, 5 µg/mL transferrin, 2 mM L-glutamine, 10^−9^ M triiodothyronine, 20 mM HEPES, 2.2% D-glucose, 2% FBS, 100 units/mL penicillin, 100 µg/mL streptomycin. For adenoviral infection, mCCD cells were cultured for three days on 12-mm Transwells (0.4-µm pore; Costar, Cambridge, MA), rinsed extensively with PBS and incubated for 1 h at 37°C on 50 µl drops of PBS containing recombinant adenoviruses and 150 µl of PBS/virus on the apical surface of the Transwell, using a total multiplicity of infection (MOI) of 125. Recombinant adenovirus encoding the constitutive expression of the tetracycline-repressible transactivator at a MOI of 50 was included in all experiments to enable doxycycline (DOX) repressible synthesis of HA-tagged PIP5KI isoforms. For endocytosis experiments adenovirus encoding pIgR was also included at a MOI of 50. Following infection, mCCD cells were incubated in complete media supplemented with 20 ng/ml DOX for 24 h. Subsequently, cells were rinsed in complete media and incubated for 16 h in the absence of DOX to allow for expression of PIP5KI constructs. Where indicated, experiments were performed using Madin-Darby canine kidney (MDCK) T23 cells, which stably express pIgR as well as the tetracycline transactivator. These cells were cultured in Minimum Essential Medium (Sigma) supplemented with 10% FBS. Filter-grown cells were infected for 1 h at 37°C with 150 µl of PBS/virus added to the apical surface of the Transwell, using a MOI of 125 for control and PIP5KIβ virus and a MOI of 10 for epsin-GFP virus. Cells were used for experiments the day after infection.

### Indirect Immunofluorescence

Cells were fixed with 4% paraformaldehyde in 100 mM sodium cacodylate pH 7.4 at 37°C for 15 min, quenched in PBS with 20 mM glycine and 75 mM NH_4_Cl for 5 min, permeabilized using 0.1% TritonX-100 in the quench solution for 10 min with gentle shaking, and blocked in PBS with 1% BSA and 0.1% saponin for 1 h at ambient temperature. Virally expressed PIP5KI isoforms were detected using anti-HA epitope tag antibody (Covance; 1∶500). Rat anti-ZO-1 hybridoma R40.76 culture supernatant was used to detect ZO-1 [52]. Rhodamine-Phalloidin (Life Technologies; 1∶500) was used to visualize actin. All secondary AlexaFluor conjugated antibodies (Life Technologies) were used at a dilution of 1∶500. The cells were mounted in ProLong Gold Antifade plus DAPI (Life Technologies) or PBS for total internal reflection fluorescence (TIRF) imaging. Confocal images were taken on a Leica SP5 confocal microscope and processed using Metamorph (Molecular Devices).

### Immunofluorescence of Rat Kidney Sections

Rat kidney cryosections (4-µm thick) were obtained from Dr. Nuria Pastor-Soler and processed as described in [53]. Briefly, sections were fixed, then rehydrated in PBS for 30 min and treated with 1% SDS for 4 min, washed with PBS and then blocked with 1% BSA. Sections were then incubated with PIP5KIβ antibody (Abnova; 1∶100) for 75 min, washed with high-salt PBS (2.7% NaCl), followed by PBS, and then incubated with secondary antibody Alexa-Flour 488 (Life Technologies; 1∶400) for 1 h. The wash steps were repeated then the sections were mounted in Vectashield (Vector labs). Slides were imaged on a Leica SP5 confocal microscope.

### Quantitation of Cellular PtdIns(4,5)P_2_ Levels

Cells were labeled with ^32^P-orthophosphate, and phospholipids were extracted and analyzed by thin layer chromatography (TLC) essentially as described in [34]. Briefly, mCCD cells plated in 12-well Transwells or on plastic were co-infected with adenoviruses encoding TA (MOI 50) and the indicated PIP5KI isoform (MOI 125). The cells were incubated for 30 min in phosphate-free DMEM and then for an additional 15 min in PBS with 1 mM MgCl_2_ prior to radiolabeling for 4 h with 40 µCi/ml ^32^P-orthophosphate. Next the cells were trypsinized, 1 ml of chloroform:methanol:1N HCl (4∶3:3) was added to the pelleted cells, and the mixture was vortexed and centrifuged at 1500 rpm for at 4°C for 7 min. The organic phase was collected and washed twice with an equal volume of MeOH:1N HCl (1∶1). Radioactivity in aliquots was counted using a scintillation counter, and equal counts/min of each sample were spotted onto oxalate-treated Silica gel 60 TLC plates (EM Science) and developed in 1-propanol:2M acetic acid (65∶35). Authentic lipid standards (Avanti Polar Lipids) were included on all plates. Radiolabeled products were visualized and quantified using a phosphorimager. The percent of total was quantified by dividing the intensity of the PtdIns(4,5)P_2_ spot relative to the total acidic phospholipids (phosphatidic acid, phosphatidylinositol, phophatidylinositol phosphate, and phosphatidylinositol 4,5-bisphosphate).

### Endocytosis of ^125^I-IgA

Human polymeric IgA (Nordic) was iodinated as described in [54], [55]. Approximately 2 mCi Na^125^I (Perkin-Elmer) was used to iodinate 40 µg of IgA in a final volume of 600 µl. mCCD cells were plated in 12-well Transwells and infected with pIgR, TA, and PIP5KIβ, PIP5KIα or PIP5KIγ adenovirus as described above. The next day each Transwell was incubated with approximately 160 ng ^125^I-IgA for 1 h in media MEM/BSA (MEM, HBSS, 0.6% BSA, 20 mM HEPES, pH 7.4) on ice, then washed extensively with ice cold media MEM/BSA to remove unbound radioligand. The cells were then incubated in pre-warmed media at 37°C for 0, 2.5, or 5 min, then rapidly chilled. To remove ^125^I-IgA from the cell surface, cells were incubated for 30 min at 4°C with 100 µg/ml L-1-tosylamide-2-phenylethylchloromethyl-ketone-treated trypsin (Sigma), then stripped with 150 mM glycine buffer, pH 2.3 for 15 min at 4°C. Filters were cut out of their plastic inserts and cell-associated radioactivity was counted using a gamma counter (PerkinElmer). Internalized ^125^I-IgA was quantitated relative to total ^125^I-IgA (recovered in the cells, trypsin and glycine strips, and the incubation medium).

### Recycling of ^125^I-IgA

Recycling assays were performed essentially as described in [56]. Briefly, mCCD cells were co-infected with pIgR, TA, and PIP5KIβ adenovirus. The following day cells were incubated with ^125^I-IgA at 37°C for 45 min in MEM/BSA. Cells were extensively washed using cold MEM/BSA on ice, then pre-warmed MEM/BSA was added to the apical and basolateral chambers and cells were kept in a 37°C water bath. At each time point the media was collected and replaced with fresh media. After the last time point the filters were removed from their inserts and radioactivity in each media and filter sample was determined using a gamma counter and the fraction of IgA recycled at each time point was quantitated..

### Protein Purification

GST fusion proteins were expressed in *E. Coli* BL21 cell cultures at 37°C until log phase (OD600 = 0.6), at which time the cultures were transferred to ambient temperature and isopropyl-1-thio-β-D-galactopyranoside (100 µM) was added. After 3 h of shaking, the culture was centrifuged at 12,000 *g* for 10 min at 4°C then stored at −80°C overnight. The frozen tubes were thawed on ice and the pellets were resuspended in sonication buffer (50 mM Tris-HCl, pH 8.0, 300 µM NaCl, 0.2% Triton X-100, 10 mM β-mercaptoethanol) with 1 mM PMSF. The cell suspension was sonicated (Fisher Scientific Sonic Dismembranator Model 100) five times for 30 sec with 30 sec breaks on ice in between. Homogenates were then centrifuged at 40,000 *g* for 20 min at 4°C and supernatants were loaded onto PBS-washed glutathione Sepharose beads and rotated end-over-end for 2.5 h at 4°C. GST proteins were eluted with 1 mL of glutathione elution buffer (25 mM Tris-HCl, pH 8.0, 250 mM NaCl, 10 mM glutathione) plus 5 µM DTT for 10 min on ice with gentle rocking. Samples were centrifuged at 400 *g* for 4 min, and the supernatants collected. The elution process was repeated a total of three times, and the eluates were combined and dialyzed in a Slide-A-Lyzer (Pierce) overnight in 1.5 L of PBS at 4°C. Fresh PBS was added the next morning and the protein was extracted and quantified 7 h later and stored at -80°C.

### Liposome Binding

Liposomes were prepared as described in [46] with 10% PtdIns(4,5)P_2_. Varying amounts of ENTH or PTB GST fusion proteins were added to 20 µl incubation buffer (25 mM HEPES–KOH, pH 7.2, 125 mM potassium acetate, 5 mM magnesium acetate, 2 mM EDTA, 2 mM EGTA, 1 mM DTT, 0.1 mg/ml BSA), 10 µl BSA (2 mg/mL), 20 µl liposomes, and dH_2_O in a final volume of 200 µl. Samples were incubated at ambient temperature for 30 min and then centrifuged at 20,000 *g* for 15 min at 4°C. Aliquots of each supernatant and pellet (2.6% and 20% of total, respectively) were electrophoresed on SDS–PAGE gels and the gels were stained with Coomassie Blue. Images of the stained gels were acquired using a Bio-Rad Gel Doc XR+ and Image Lab software was used to measure the intensity of the supernatant and pellet fractions. The total protein recovered at each concentration was normalized to a standard curve generated from the intensities of the supernatant plus pellet fractions (each normalized to total input). To determine percent of protein bound to liposomes, the intensity of the normalized pellet fraction was divided by the normalized total protein values.

### Total Internal Reflection Fluorescence Imaging

MDCK cells were grown on permeable supports and processed for immunofluorescence described above. The filters were cut out of their inserts and inverted onto a coverslip. Cells were imaged using a Nikon Eclipse Ti automated inverted microscope outfitted for total internal reflection fluorescence (TIRF) imaging and a 100x 1.49 NA TIRF objective. Solid-state lasers of 488, 561, and 647 nm were used as light sources. Images were acquired with an iXon+897 EM-CCD camera driven by iQ (Andor LLC). The depth of field of illumination was about 150 nm. Imaris (Bitplane Scientific Software) was used to quantitate the number of epsin spots per cell. The expected spot size was set to 0.3 µm and the threshold was set automatically to determine the number of spots in each frame. The area of each cell was determined using ImageJ software (NIH) to calculate the number of spots per square micron.

### Electron Microscopy

MDCK cells cultured on filters were infected with control or PIP5KIβ adenovirus then fixed in 2.0% (v/v) glutaraldehyde and 2.0% (v/v) paraformaldehyde in 100 mM sodium cacodylate buffer, pH 7.4 for 30 min at 37°C. The tissue was then post-fixed in reduced 1.5% (v/v) OsO_4_, *en bloc* stained overnight in 0.5% (v/v) uranyl acetate, dehydrated in alcohol, and embedded in epon. The tissue was sectioned using a diamond knife (Diatome USA, Hatfield, PA), and sections, silver in color, were stained with lead citrate, viewed in a JEOL 100CX transmission electron microscope, and images acquired using an L9C Peltier-cooled TEM camera system (Scientific Instruments and Applications, Inc.; Duluth, GA). Representative digital images were imported into Adobe Photoshop CS4 (Adobe Systems Inc.; San Jose, CA), the contrast was corrected, and composite images were generated using Adobe Illustrator CS4 (Adobe Systems Inc.).

To quantify the number of clathrin-coated structures per µm of apical or basolateral membrane we used our previously described methods [57]. Briefly, random epon blocks were sectioned perpendicular to the length of the epithelium to obtain vertical sections. At least 35 randomly chosen cross sections of MDCK apical or basolateral membrane were photographed at 27,000 X and then printed. For each image a grid of cycloidal arcs [see grid C3 58]] was placed over the image and the number of intersections of plasma membrane (*I_P_*) with the grid, and the number of the following clathrin-coated structures (*Q_CCS_*) was recorded: those with only a small amount of membrane curvature (shallow coated pits); those with a significant curvature, but lacking a discernable neck (invaginated pit); those with significant curvature and attached to the surface by a neck structure (deeply invaginated pits), and coated vesicles within 1 µm of the surface. The following formula was used to estimate the boundary length of apical or basolateral plasma membrane in each image (B_Lp_) [59]:
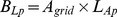


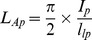
where A_grid_ is equal to the calibrated area of the cycloid grid, which was 32.96 µm^2^ in our experiments, L_Ap_ is equal to the length of plasma membrane per unit area (units of µm/µm^2^), and l_lp_ is equal to the calibrated length of line probe, which was 44.4 µm in our experiments. To estimate the number (Q) of each of the clathrin-coated structures (CCS) per length of plasma membrane the following formula was used:



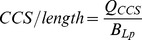



## Results

### PIP5KI Isoform Localization, Expression, and Activity in Kidney Cells

**Figure 1:**
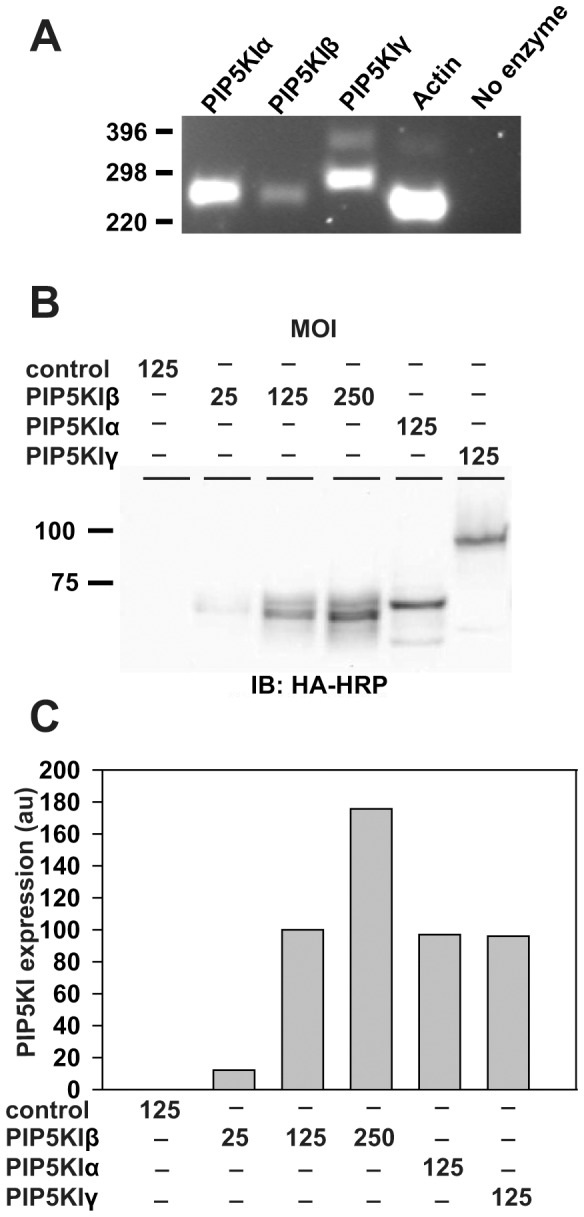
Endogenous and adenovirus-mediated expression of PIP5KI isoforms in renal epithelial cells. (A) Reverse-transcriptase PCR of mRNA isolated from mCCD cells was performed to detect expression of endogenous PIP5KI isoforms. Actin was amplified as a positive control, and actin primers were included in the sample with no enzyme. Predicted sizes are 246 (PIP5KIα), 247 (PIP5KIβ), 276 (PIP5KIγ), and 231 bases (actin). (B) Polarized mCCD cells were mock-infected or infected with adenoviruses encoding HA-tagged PIP5KIα, PIP5KIβ, or PIP5KIγ isoforms at the indicated multiplicity of infection (MOI). Cell lysates were harvested and western blotted using anti-HA antibody to detect PIP5KI expression. The expected masses are approximately 68 (PIP5KIα and PIP5KIβ) and 91 kDa (PIP5KIγ). (C) Densitometric quantitation of the western blot above using arbitrary units (au) normalized to PIP5KIβ MOI 125 demonstrates that PIP5KIβ expression at different MOIs is linear and that expression levels of all three isoforms at MOI 125 is similar.

All three isoforms of PIP5KI are known to be expressed in mammalian kidney [6], [7], [8], [10]. To confirm that they are also expressed endogenously in immortalized murine cortical collecting duct (mCCD) cell lines, we amplified mRNA isolated from these cells using primers specific for each isoform. As shown in Figure 1A, message for all three isoforms could be readily detected in these cells by RT-PCR.

To examine the distribution and role of PIP5KI isoforms in mCCD cells, we generated DOX-repressible replication-deficient adenoviruses expressing each PIP5KI isoform tagged C-terminally with the HA epitope. mCCD cells were co-infected with varying MOIs of adenovirus together with adenovirus constitutively expressing the tetracycline transactivator, which is required for expression of PIP5KIs driven by the tet operon. Cells were incubated with 20 ng/ml DOX overnight to allow cell repolarization following infection in the absence of viral expression, and then DOX was washed out overnight to enable PIP5KI expression. Cells were solubilized and blotted with anti-HA antibody to compare protein expression. The blot was imaged on a VersaDoc (Bio-Rad) imaging system and the bands were quantitated using Quantity One (Bio-Rad) software. As shown in Figure 1B, PIP5KIβ expression was linear with viral MOI over the tested range of 25–250. Moreover, all PIP5KI isoforms showed similar expression levels when infected with the same MOI based on the densitometric analysis of the HA-tagged protein intensities (Figure 1C).

**Figure 2:**
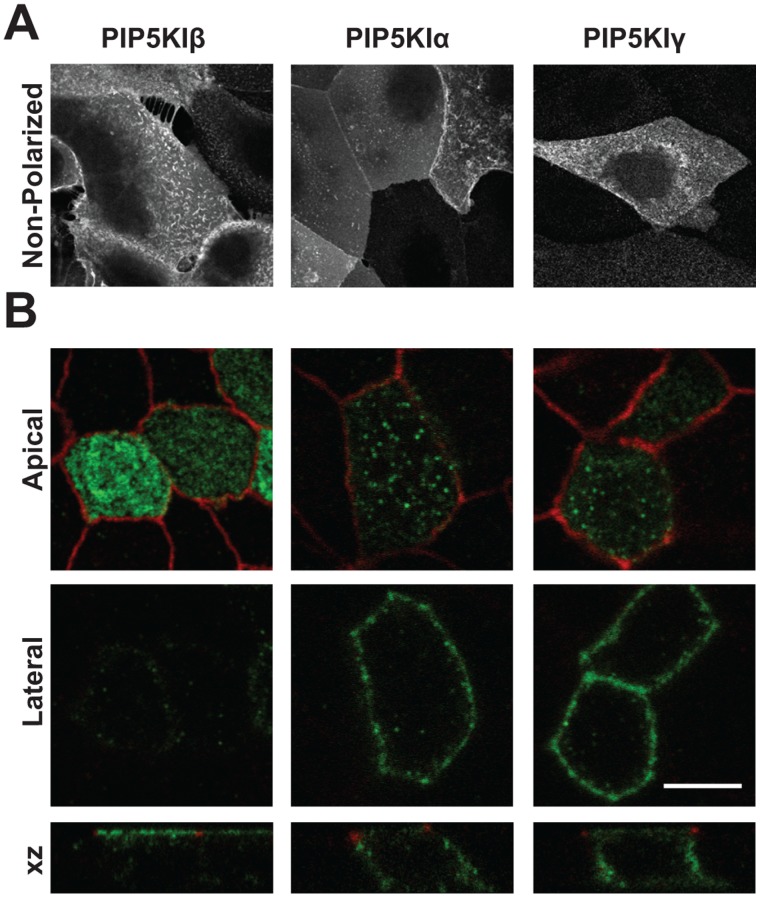
Localization of HA-tagged PIP5KI isoforms in polarized mCCD cells. Mouse cortical collecting duct cells grown on (A) coverslips (non-polarized) or on (B) permeable supports were infected with adenovirus encoding PIP5KIβ, PIP5KIα or PIP5KIγ and processed for indirect immunofluorescence to visualize PIP5KIs using anti-HA epitope antibody [green in (B)]. Anti-ZO-1 antibody was included in filter-grown cells (red) to mark tight junctions. All isoforms are localized to the plasma membrane as well as intracellular punctate structures in non-polarized cells. In contrast, PIP5KI isoforms are differentially distributed in polarized cells. Confocal sections acquired at the apical and lateral regions of cells are shown, as well as an xz reconstruction of the entire confocal stack. Whereas PIP5KIα and PIP5KIγ are found in a non-polarized or lateral distribution, PIP5KIβ localizes almost exclusively at the apical surface. Scale bar: 10 µm.

The subcellular localization of HA-tagged PIP5KI isoforms in mCCD cells was examined by indirect immunofluorescence. In non-polarized cells, all three isoforms localized largely to the cell surface and internal structures, consistent with previous reports [13], [14], [60] (Figure 2A). In contrast, in polarized cells grown on permeable supports, PIP5KI isoforms were differentially distributed to distinct surface domains. As we previously observed [34], [48], PIP5KIβ was strikingly localized to the apical surface (Figure 2B). In contrast, PIP5KIα and PIP5KIγ are found primarily at the basolateral domain with minimal localization to the apical domain (Figure 2B). This is consistent with a previous study that reported a lateral distribution of endogenous PIP5KIγ in MDCK cells [11]. These differences in the localization of PIP5KIs in polarized cells suggested that individual isoforms might differentially regulate PtdIns(4,5)P_2_ synthesis at the apical and basolateral domains.

**Figure 3:**
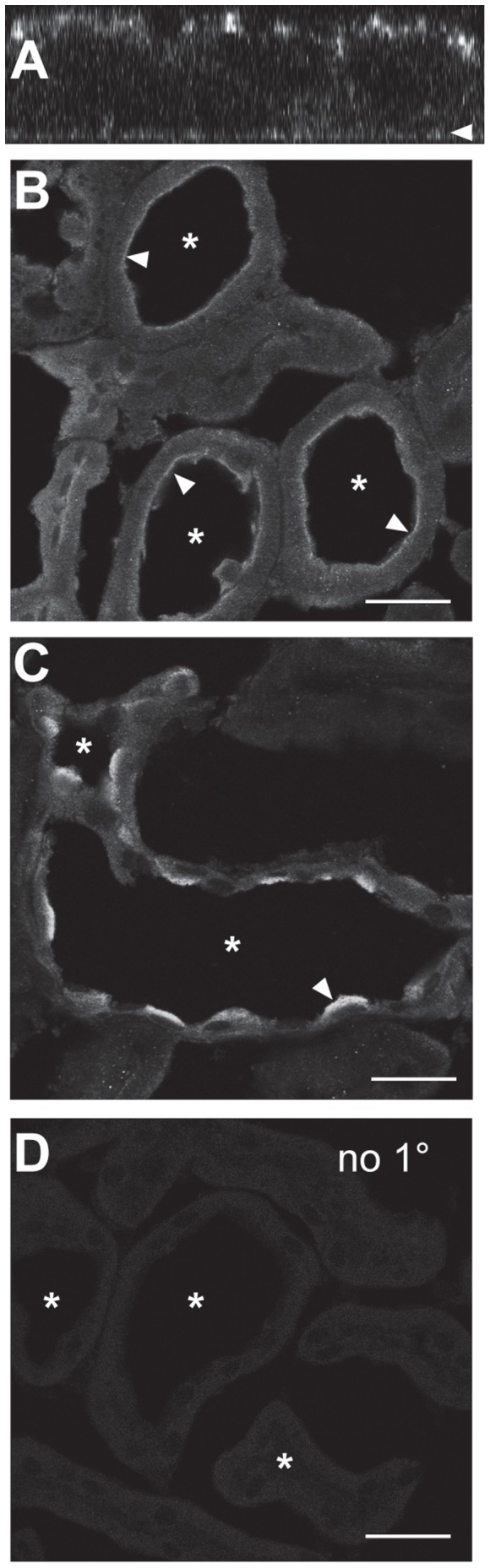
Endogenous PIP5KIβ localizes to the apical surface of kidney cells. (A) Mouse cortical collecting duct (mCCD) cells cultured on permeable supports were fixed and processed for indirect immunofluorescence to detect endogenous PIP5KIβ, which localizes predominately to the apical surface. The arrowhead denotes the position of the filter. (B-C) 4 µm rat kidney cortex slices were fixed and stained to detect PIP5KIβ. Tubule lumens are indicated by asterisks. PIP5KIβ is enriched at the apical (lumenal) surface, indicated by an arrowhead, of (A) kidney proximal tubule and (B) collecting duct cells. (D) A kidney section processed identically but without primary antibody shows minimal staining. Scale bar: 25 µm.

We also assessed the localization of endogenous PIP5KIβ to determine whether the apical distribution of HA-tagged PIP5KIβ reflected the true distribution of this isoform in polarized kidney cells. As shown in Figure 3A, endogenous PIP5KIβ in mCCD cells was also preferentially distributed to the apical surface, consistent with our results using virally expressed PIP5KIβ. Similarly, endogenous PIP5KIβ was visualized in rat kidney cortex sections. In the kidney, the apical surface of cells faces the lumen, whereas the basolateral surface faces the interstitium. We found that in the rat kidney cortex sections PIP5KIβ was concentrated at the apical (lumenal) surface of proximal tubules and cortical collecting duct cells (Figure 3B and 3C, respectively).

**Figure 4:**
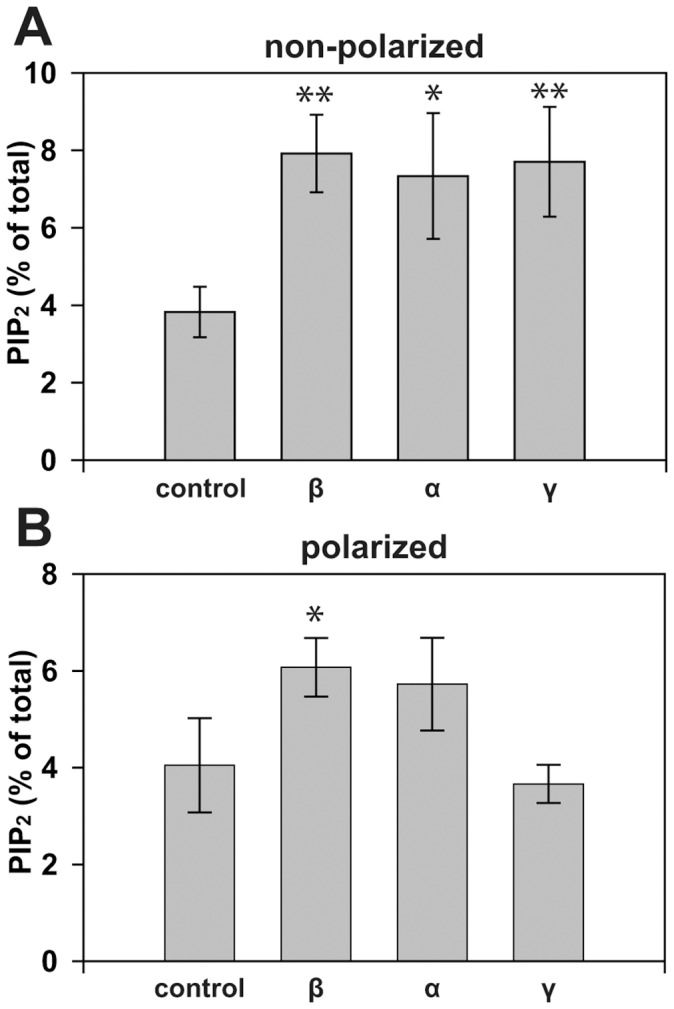
Effect of PIP5KI overexpression on PtdIns(4,5)P_2_ levels in renal epithelial cells. Non-polarized (A) or polarized (B) mCCD cells infected with either control adenovirus or virus encoding PIP5KIβ, PIP5KIα, or PIP5KIγ were radiolabeled with ^32^P_i_ for 4 h then acidic lipids collected and analyzed by TLC. PtdIns(4,5)P_2_ was quantified as the percent of total acidic phospholipids recovered in each sample. Each graph represents the mean +/− SE of at least four experiments performed in duplicate. One way ANOVA with Bonferroni correction was performed to determine statistical significance (**p<0.01 *p<0.05). Whereas expression of each isoform increased cellular PtdIns(4,5)P_2_ levels in non-polarized cells, only PIP5KIβ had a significant effect on PtdIns(4,5)P_2_ in polarized cells.

We performed thin-layer chromatography (TLC) to test whether cells overexpressing the various PIP5KI isoforms had elevated levels of PtdIns(4,5)P_2_. mCCD cells grown on plastic or permeable supports were infected with adenoviruses encoding TA and PIP5KI isoforms, radiolabeled with ^32^Pi, and anionic phospholipids were isolated and analyzed by TLC. Expression of each of the three isoforms significantly increased PtdIns(4,5)P_2_ levels in non-polarized cells (Figure 4A), confirming that the adenovirally-expressed enzymes are active. In contrast, only PIP5KIβ expression significantly increased PtdIns(4,5)P_2_ levels in polarized mCCD cells. PtdIns(4,5)P_2_ levels in cells overexpressing PIP5KIα also had a consistent tendency to increase, though this was not statistically significant over four experiments (Figure 4B). These data suggest that the PIP5KI isoforms have access to distinct pools of PtdIns(4,5)P_2_ in polarized epithelial cells, consistent with their differential localizations to membrane domains.

### Overexpression of PIP5KIβ Selectively Stimulates Apical Endocytosis

**Figure 5:**
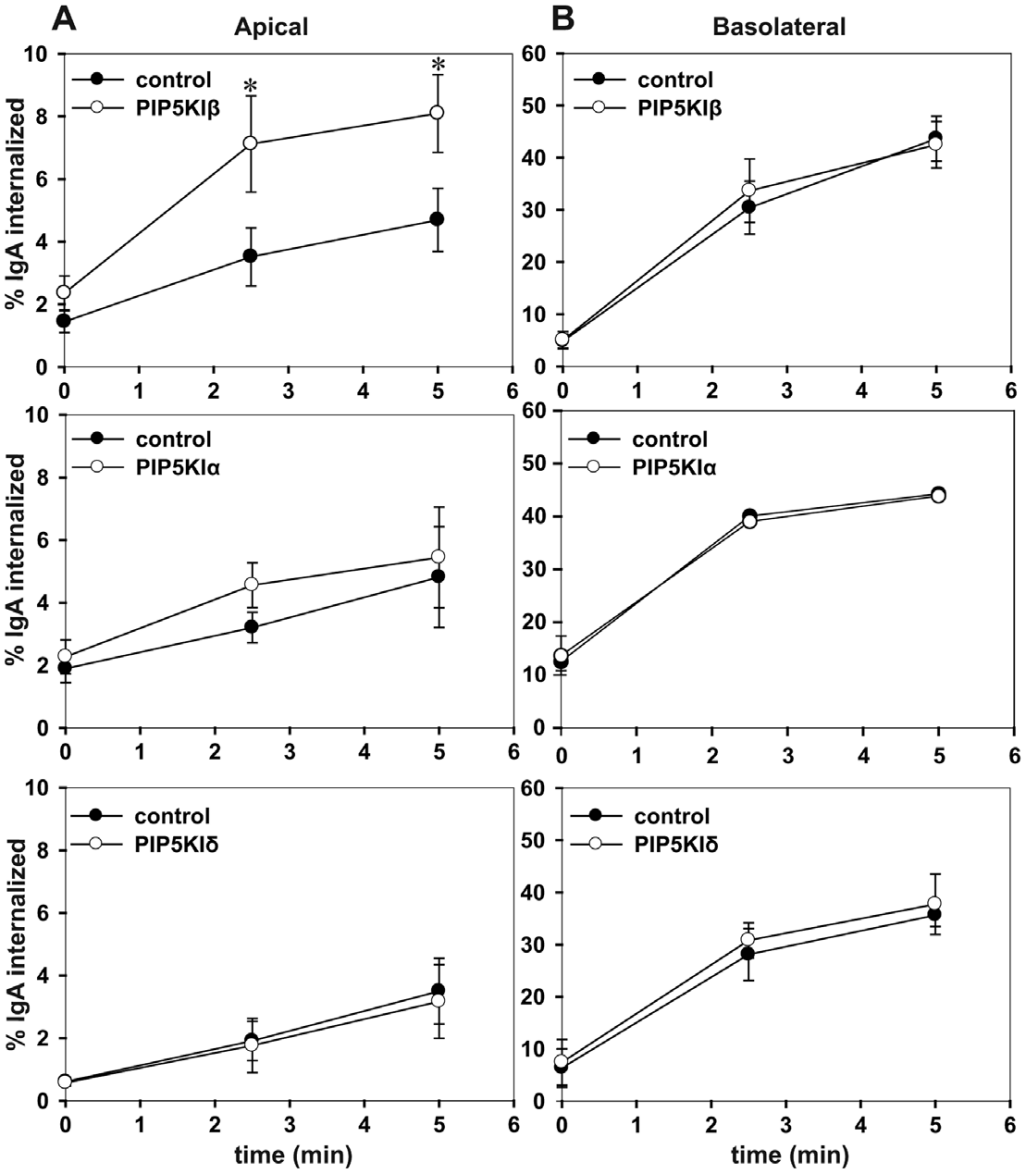
Apical endocytosis is selectively increased in cells overexpressing PIP5KIβ. Internalization kinetics of ^125^I-IgA from the apical (A) or basolateral (B) surface were assessed in polarized mCCD cells co-infected with adenovirus encoding the polymeric immunoglobulin receptor and either control adenovirus or adenovirus expressing PIP5KIβ, PIP5KIα, or PIP5KIγ. The graphs shows the percent of ^125^I-IgA internalized over a five min time course. Each graph represents the mean +/− SE of at least three independent experiments performed in duplicate. *p<0.05 by paired t-test.

Each of the three PIP5KI isoforms has been implicated in endocytosis in various non-polarized cell types [11], [13], [14], [60]. Because PIP5KI isoforms are differentially distributed in polarized cells, we sought to determine whether overexpressing individual isoforms would differentially affect endocytosis from the apical and basolateral domains. To this end, we measured the endocytosis of the polymeric immunoglobulin receptor (pIgR), which can be internalized from both plasma membrane domains of polarized cells [61]. pIgR is synthesized in the endoplasmic reticulum, and transported through the Golgi complex, and then delivered to the basolateral surface before it is transcytosed to the apical surface [62]. pIgR also recycles from both the apical and basolateral domains, so it can be used to study endocytosis at both surfaces [63]. mCCD cells co-expressing pIgR and either PIP5KIα, PIP5KIβ, or PIP5KIγ were incubated with apically- or basolaterally-added ^125^I-IgA and endocytosis of pre-bound IgA was monitored for 0–5 min. Overexpression of PIP5KIβ significantly and reproducibly stimulated the endocytosis of pIgR from the apical surface, but had no effect on basolateral endocytosis (Figure 5). In contrast PIP5KIα and PIP5KIγ had no effect on either apical or basolateral internalization of pIgR (Figure 5). These data suggest that endocytosis and PtdIns(4,5)P_2_ synthesis at the apical surface of polarized cells is regulated primarily by PIP5KIβ.

**Figure 6:**
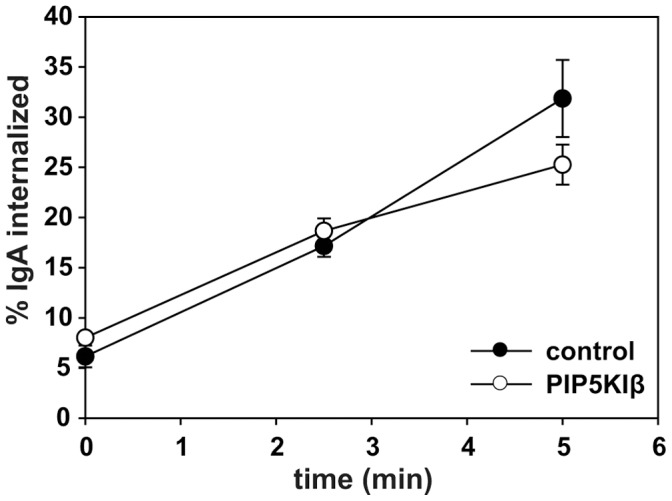
Endocytosis in non-polarized cells is unaffected by PIP5KIβ. (A) Internalization kinetics of ^125^I-IgA were measured in non-polarized cells co-infected with adenovirus encoding the polymeric immunoglobulin receptor and either control or PIP5KIβ adenovirus. The graph shows the percent of internalized ^125^I-IgA over a five min time course. The mean +/− range from one experiment performed in duplicate is shown. Similar results were obtained in three independent experiments.

The stimulation we observed upon overexpression of PIP5KIβ suggests the possibility that production of PtdIns(4,5)P_2_ is rate limiting for apical but not basolateral endocytosis. Apical endocytosis is known to proceed more slowly than basolateral endocytosis of many proteins that can be internalized from either membrane domain [33], [34], [35], [36], [37]. Elevated levels of PtdIns(4,5)P_2_ may selectively enhance recruitment of endocytic machinery to the apical surface of polarized cells and/or alleviate steric barriers to endocytosis. However, it is also possible that PIP5KI isoforms do not elevate basolateral PtdIns(4,5)P_2_ levels sufficiently to affect endocytic kinetics. The apical targeting signal in PIP5KIβ is unknown and we spent considerable effort without success attempting to mislocalize PIP5KIβ to the basolateral surface in order to test this directly. As an alternative approach, we tested whether overexpression of PIP5KIβ stimulated endocytosis of pIgR in non-polarized cells, where changes in cellular PtdIns(4,5)P_2_ levels were clearly evident. Non-polarized cells maintain a rapid rate of constitutive pIgR endocytosis similar to the basolateral endocytic rate for this protein in polarized cells. As shown in Figure 6, endocytic kinetics of pIgR in non-polarized cells were not affected by overexpression of PIP5KIβ. Because PtdIns(4,5)P_2_ has also been implicated in regulating exocytosis [48], [64], [65], [66], [67], [68] we also tested whether overexpression of PIP5KIβ affects pIgR recycling. As shown in Figure S1, we found no differences in either apical or basolateral pIgR recycling rates in cells overexpressing PIP5KIβ compared with control. We conclude that PtdIns(4,5)P_2_ is rate limiting for apical but not basolateral endocytosis, and that elevation of PtdIns(4,5)P_2_ levels does not appreciably stimulate endocytosis in non-polarized cells.

### Increased Cellular PtdIns(4,5)P_2_ Increases the Maturation Rate of Apical CCPs

**Figure 7:**
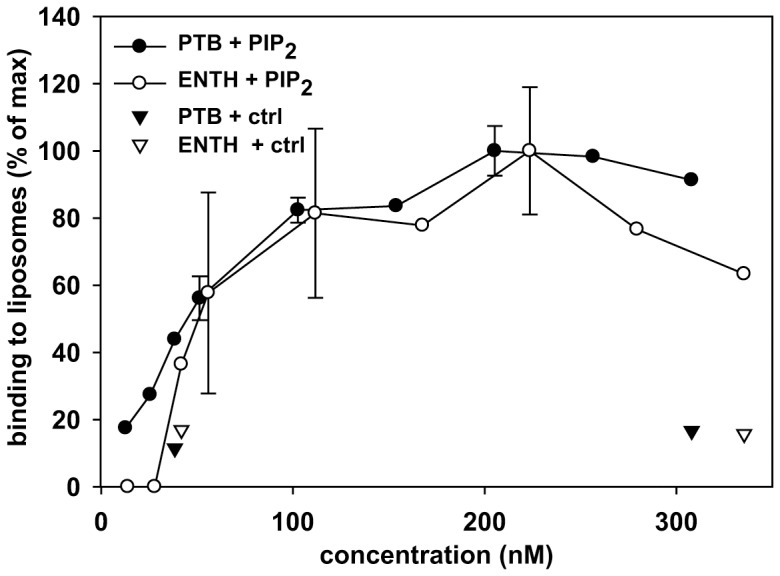
Recombinant ENTH and PTB domains bind to PtdIns(4,5)P_2_-containing liposomes with similar affinities. Increasing concentrations of PTB-GST or ENTH-GST (the PtdIns(4,5)P_2_ binding domains of Dab2 and epsin, respectively) were incubated with control or PtdIns(4,5)P_2_-containing liposomes at ambient temperature for 30 min. The liposomes were pelleted and aliquots of the supernatant and pellet were analyzed by Coomassie staining after SDS-PAGE. The protein recovery in the pellet was quantified by scanning densitometry. Neither domain bound significantly to control liposomes (triangles), and there is no significant difference in the dose dependence of ENTH or PTB binding to PtdIns(4,5)P_2_-containing liposomes (circles). Data represent the combined results from three independent experiments performed using different protein concentrations. The mean +/− SE or range is plotted for data from multiple experiments.

PIP5KIβ may stimulate apical endocytosis generically, or could selectively enhance uptake of cargoes in an adaptor-selective manner. There is conflicting evidence for adaptor-selective effects of PtdIns(4,5)P_2_ on endocytosis. Overexpression of PIP5KIβ was previously found to decrease ENaC currents in mCCD cells, and also decreased surface expression of the channel in *Xenopus* oocytes, suggesting that increased PtdIns(4,5)P_2_ stimulates endocytosis of this epsin-dependent channel [34]. In the same study, surface expression and current of the ARH-dependent channel ROMK were slightly elevated, suggesting that the stimulatory effect of PIP5KIβ on channel endocytosis may be selective for epsin-dependent cargoes [69]. However, overexpression of PIP5KIβ stimulated endocytosis of another ARH/Dab-2-dependent apical protein, megalin, in HK-2 cells [70], [71], [72]. Because epsin and ARH/Dab-2 bind to PtdIns(4,5)P_2_ via ENTH and PTB domains, respectively, we asked whether these domains might engage PtdIns(4,5)P_2_ with differing affinities. To this end, we purified recombinant PtdIns(4,5)P_2_ binding domains of Dab2 and epsin and measured the relative affinity of these to PtdIns(4,5)P_2_-containing liposomes using a sedimentation assay. As expected, both the ENTH domain and the PTB domain bound better to PtdIns(4,5)P_2_-containing liposomes than to control liposomes lacking PtdIns(4,5)P_2_ (Figure 7). Both domains had similar affinities for PtdIns(4,5)P_2_, (∼50 nM). The ENTH domain was previously reported to have a 23 nM affinity for PtdIns(4,5)P_2_ containing liposomes [73]. While the adaptor protein binding dose response curve showed apparently saturating kinetics, a significant fraction (60%) of the added ENTH protein was recovered in the supernatant, even at the highest concentrations added (Figure S2). When the unbound material in the supernatant was collected and tested for rebinding to liposomes, it bound with the same affinity as the original preparation (not shown). This result suggests that PIP5KIβ-mediated increases in PtdIns(4,5)P_2_ levels are unlikely to cause differential recruitment of distinct adaptors to the apical surface and instead have a general stimulatory effect on apical endocytosis kinetics.

**Figure 8:**
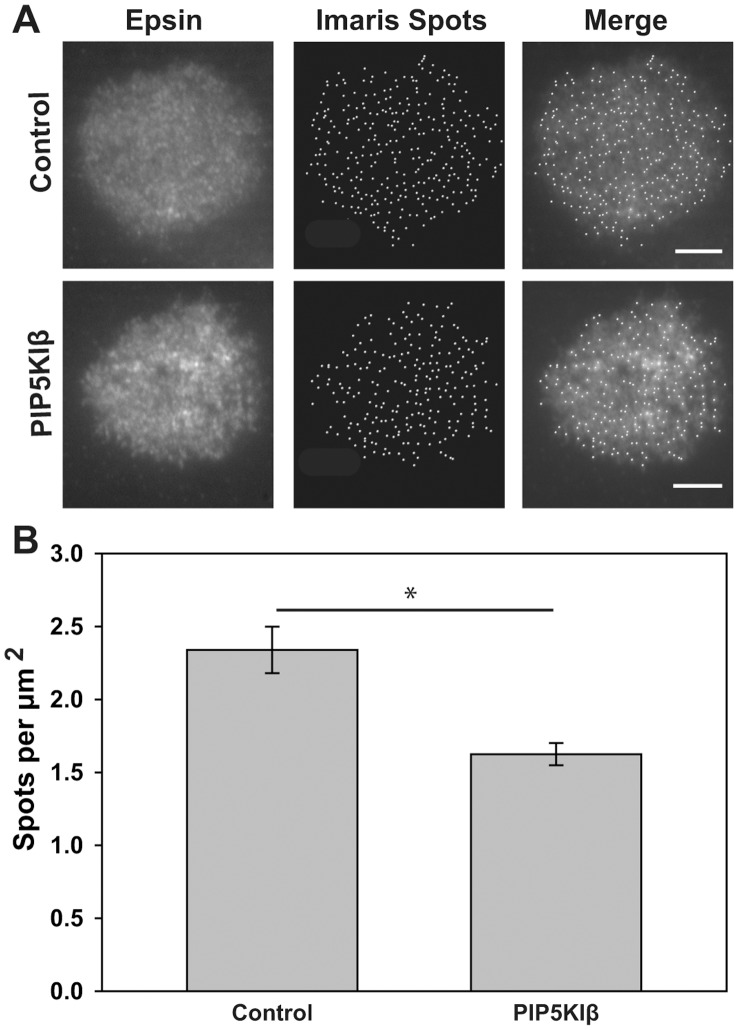
There are fewer apical CCPs in MDCK cells overexpressing PIP5KIβ. MDCK cells were cultured on permeable supports and co-infected with adenovirus encoding epsin-GFP and either PIP5KIβ or control virus. Filters were fixed and processed to detect HA-tagged PIP5KIβ in order to determine which cells were overexpressing the kinase, then mounted in PBS for TIRF microscopy. (A) TIRF images of epsin-GFP in control and PIP5KIβ overexpressing cells are shown with corresponding detection of spots by Imaris. Scale bar: 5 µm. (B) Imaris analysis of TIRF data (n = 23 cells from three experiments) indicates that there is a decrease in epsin spots per square micron upon over expression of PIP5KIβ. Student’s t-test was performed to assess statistical significance (*p<0.001).

The stimulation of apical endocytosis by PIP5KIβ could be due to an increase in the number of apical CCPs or an increase in the maturation rate of individual apical CCPs. We therefore assessed whether either of these contributed to enhanced apical endocytosis in PIP5KIβ overexpressing cells. We used TIRF microscopy to determine the number of CCPs at the apical surface of MDCK cells overexpressing PIP5KIβ compared with control. MDCK cells were grown on filters and co-infected with adenovirus encoding epsin-GFP and either control adenovirus or adenovirus encoding PIP5KIβ. Epsin is known to colocalize with the vast majority (97%) of CCPs in HeLa cells [74]. The cells were fixed, stained with anti-HA antibody to detect PIP5KIβ, and the filter was cut out and placed in a machined chamber containing an adjustable piston that positions the filter firmly against a coverslip for TIRF illumination. Epsin-GFP spots were identified and quantitated using Imaris software. Epsin-GFP spots did not colocalize with actin or with a diffuse cytosolic marker (RFP-tagged FKBP5), confirming that the spots represented CCPs rather than aberrations in the apical plasma membrane (not shown). As shown in Figure 8, the number of epsin-GFP spots detected was significantly decreased in cells overexpressing PIP5KIβ compared with control. These results suggest that the increase in the apical endocytosis of IgA upon overexpression of PIP5KIβ is not due to increased numbers of CCPs.

**Figure 9:**
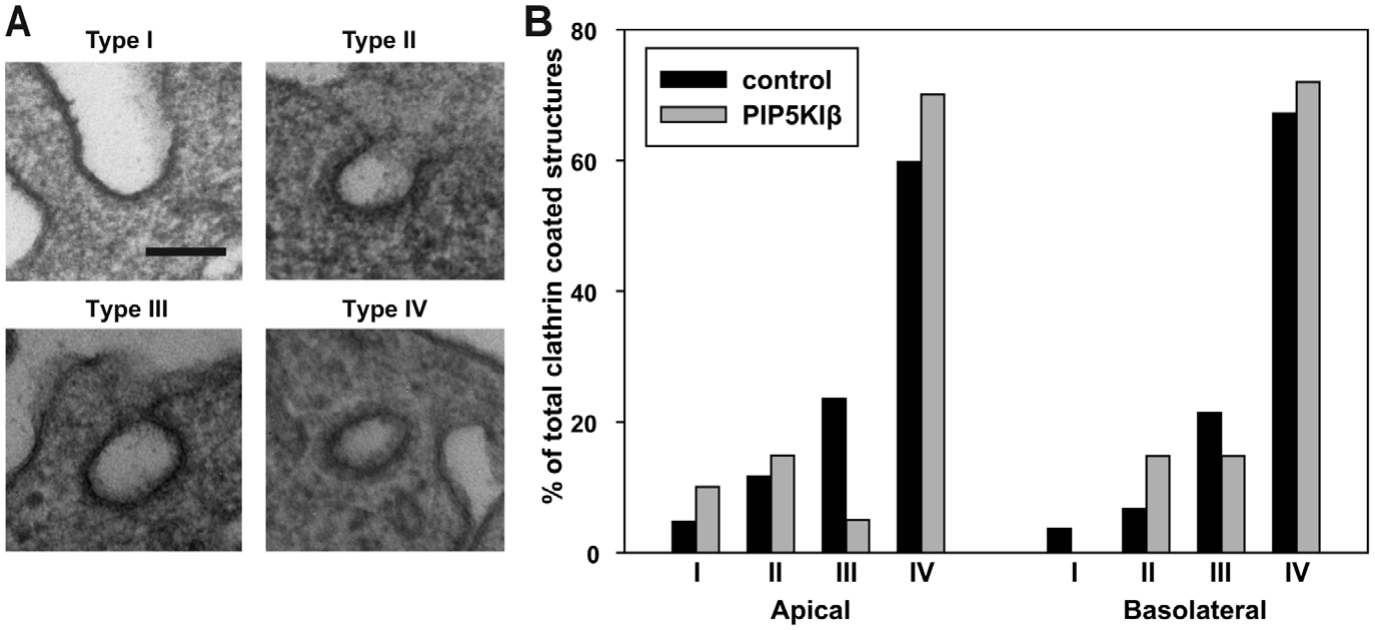
Apical CCP maturation is enhanced in PIP5KIβ-overexpressing cells. MDCK cells cultured on permeable supports and infected with control adenovirus or adenovirus encoding PIP5KIβ were processed for electron microscopy. (A) Representative EM images show each of the stages of CCP maturation that were quantitated for analysis. Scale bar: 100 nm. (B) Analysis of data from 33 control cells and 35 PIP5KIβ-overexpressing cells shows a decrease in the frequency of type III CCPs upon overexpression of PIP5KIβ.

To quantitate CCP maturation, we fixed control or PIP5KIβ-overexpressing polarized MDCK cells and processed them for electron microscopy (EM). Blocks were sectioned perpendicular to the plane of the filter to visualize the apical and basolateral cell surfaces, and CCPs and vesicles identified and quantified as described in Methods. CCPs were classified as shallow (type I), invaginated (type II), or deeply invaginated (type III). Clathrin-coated vesicles within 1 micron of the membrane were classified as fully budded (type IV). Examples of each class and the distribution of apical and basolateral coated pits and vesicles within these classes is shown in Figure 9A. Consistent with previous observations [75] we observed approximately 1.5 times more apical than basolateral CCPs per unit length of membrane in control cells (Table S1), however, the total number of pits identified was low, precluding rigorous statistical analysis. Nevertheless, the most striking difference we observed was a decrease in mature (Type III) pits at the apical surface of PIP5KIβ-overexpressing cells compared with control cells (Figure 9B). In combination with our TIRF data, this result is consistent with the interpretation that an increased rate of budding of apical CCPs may account for the stimulation of apical endocytosis we observed upon overexpression of PIP5KIβ in polarized kidney cells.

## Discussion

Precise spatially and temporally regulated synthesis and conversion of phosphatidylinositol species is essential for their myriad functions in cellular homeostasis. Here we find that the three isoforms of PIP5KI, which together synthesize the majority of PtdIns(4,5)P_2_ in cells, are differentially distributed along the plasma membrane of polarized kidney cells and have access to distinct pools of substrate. Overexpression of PIP5KIβ, which is largely confined to the apical domain, selectively stimulated apical endocytosis of cargo. In contrast, basolateral endocytosis kinetics, which are considerably faster than apical internalization rates, were unaffected by expression of any isoform. Imaging studies using TIRF and EM suggest that the overexpression of PIP5KIβ increases the maturation efficiency of CCPs to generically stimulate apical internalization kinetics. Thus, availability of PtdIns(4,5)P_2_ appears to be a rate limiting factor for apical endocytosis. This finding has implications for our understanding of how apical endocytosis is regulated in response to physiological cues.

The localization of PIP5KIβ to the apical surface of mCCD cells and rat kidney tissue is consistent with previous reports which showed that overexpressed PIP5KIβ has an apical distribution in polarized kidney cells [11], [34], [48]. PIP5KIγ localizes to the basolateral surface in polarized kidney cells, consistent with previous reports [11], as does PIP5KIα. While the signals that determine the distinct distributions of PIP5KI isoforms are not known, the differential localization of these enzymes suggests that they have different and non-redundant functions in polarized cell trafficking and/or signaling. In support of this, whereas overexpression of each of the three PIP5KI isoforms increased PtdIns(4,5)P_2_ levels in non-polarized kidney cells, only PIP5KIβ overexpression significantly affected PtdIns(4,5)P_2_ levels in polarized cells. This suggests that PIP5KIα and PIP5KIγ cannot access the same substrate pools as PIP5KIβ. This was not a surprising observation, given that overexpressed PIP5KIγ and PIP5KIα tend to localize to the basolateral domain of polarized mCCD cells. Thus, PIP5KIβ appears to be the primary enzyme responsible for synthesis of the apical PtdIns(4,5)P_2_ pool in mCCD cells.

The concept that PIP5KI isoforms synthesize functionally distinct pools of PtdIns(4,5)P_2_ is not new [76]. As evidence of this, PIP5KIγ knockout (KO) reduced PtdIns(4,5)P_2_ levels in the mouse brain by 40%, whereas PIP5KIα or PIP5KIβ KO had no effect [65], [77]. In contrast, there was no change in PtdIns(4,5)P_2_ in bone marrow macrophages derived PIP5KIγ KO mice, but there was a decrease in PtdIns(4,5)P_2_ in PIP5KIα or PIP5KIβ KO macrophages [78], [79]. Additionally, overexpression of PIP5KIβ in HeLa cells led to an increase in PtdIns(4,5)P_2_ levels, whereas expression of PIP5KIα and PIP5KIγ had no effect on PtdIns(4,5)P_2_ [14]. Moreover, PIP5KIγ appears to be selectively required for localized synthesis of PtdIns(4,5)P_2_ in the uropod of primary neutrophils [80]. Thus, the ability of PIP5KI isoforms to access distinct pools of PtdIns(4)P_2_ is apparently cell-type specific.

The requirement for PtdIns(4,5)P_2_ during multiple steps of the endocytic process has long been known [81], and several groups have confirmed that rapid depletion of PtdIns(4,5)P_2_ upon acute targeting of PtdIns(4,5)P_2_ 5′-phosphatase domains profoundly inhibits endocytosis [28], [29], [30]. However the role of individual PIP5KIs in modulating endocytosis in non-polarized cells remains unclear and data from different laboratories or approaches are not necessarily congruent. Overexpression of PIP5KIγ in experiments by Bairstow et al. enhanced transferrin uptake in MDCK cells grown on plastic [11], whereas another group reported that overexpression of either PIP5KIα or PIP5KIβ but not PIP5KIγ stimulated transferrin uptake and increased CCP number in CV1 cells [14]. The same study found that siRNA mediated knockdown of PIP5KIβ (but not PIP5KIα or PIP5KIγ) in HeLa cells inhibited transferrin internalization kinetics [14]. In contrast, a more recent study found that overexpression of PIP5KIα slightly inhibited transferrin endocytosis while paradoxically increasing CCP initiation and size [82]. In yet another study, truncation of the kinase domain of PIP5KIα but not PIP5KIβ inhibited endocytosis of EGF receptors in NR6 cells [60]. These discrepancies could arise from differences in cell type, changes in expression of endogenous isoforms upon overexpression, or knockdown of the others [11], [14]. Alternatively, constitutive endocytosis kinetics or substrate levels may differ between cell types.

We found that overexpression of PIP5KIβ but not PIP5KIα or PIP5KIγ stimulated apical endocytosis of IgA via the pIgR, whereas none of these enzymes altered basolateral internalization kinetics of the same cargo. This result is consistent with our observation that despite their partial localization to the apical surface, overexpression of PIP5KIα or PIP5KIγ had no significant effect on cellular PtdIns(4,5)P_2_ levels in polarized cells. Because overexpression of PIP5KIβ increased PtdIns(4,5)P_2_ levels but had no effect on the kinetics of pIgR endocytosis in non-polarized cells, we conclude that PtdIns(4,5)P_2_ levels may be rate limiting at the apical surface. Endocytosis in non-polarized cells is similar to endocytosis at the basolateral surface as it has rapid kinetics and is relatively unaffected by actin depolymerizing reagents [38]. We hypothesize that increased PtdIns(4,5)P_2_ has a generic effect on apical internalization because (1) internalization of several other cargoes that engage distinct adaptors is apparently stimulated in cells overexpressing PIP5KIβ [34], [70] and (2) we found no difference in the affinity of adaptor protein PtdIns(4,5)P_2_-binding domains for PtdIns(4,5)P_2_-containing liposomes.

Many laboratories have observed that endocytosis of the same cargo protein proceeds more slowly from the apical than the basolateral surface of polarized cells, suggesting that clathrin mediated endocytosis may be constrained by morphological features of the apical surface [33], [34], [35], [36], [37]. Apical CCP formation is confined to the base of microvilli, and the lipid composition or high membrane curvature at that site could impede membrane deformation or remodeling necessary for CCP invagination. Indeed, Roth and colleagues previously showed that CCP maturation proceeds more slowly at the apical surface compared with the basolateral domain [35]. Moreover, the local concentration of actin within the subapical terminal web and in microvilli may impede recruitment of endocytic machinery components or vesicle formation or movement. Kirchhausen and colleagues recently demonstrated that the tension at the apical membrane is the reason for the actin-dependence of apical endocytosis [38]. PIP5KIβ-mediated changes in apical PtdIns(4,5)P_2_ levels could generically stimulate apical endocytosis by alleviating several of these impediments. Locally elevated levels of PtdIns(4,5)P_2_ could enhance binding of endocytic components to apical CCPs. Alternatively or in addition, dynamin-mediated fission or clathrin-coated vesicle transport through the actin-rich terminal web might be stimulated [45], [83]. Vesicle transport could be promoted through an increase in actin polymerization or myosin recruitment. Our studies do not identify whether one or multiple steps in endocytosis are affected upon PIP5KIβ overexpression, but support the notion that CCP maturation occurs more rapidly under these conditions. Apical TIRF imaging revealed a significant decrease in the number of apical CCPs in PIP5KIβ overexpressing cells. Additionally, our EM analysis suggested that PIP5KIβ overexpression leads to a decrease in mature pits and an increase in budded pits at the apical surface, consistent with our TIRF findings. Further studies will be required to determine which step(s) of endocytosis is stimulated upon increased PtdIns(4,5)P_2_ levels.

## Supporting Information

Figure S1 **IgA Recycling is unaffected by PIP5KIβ.** Recycling of apically-internalized ^125^I-IgA was quantified in polarized mCCD cells infected with adenovirus encoding the polymeric immunoglobulin receptor and either control or PIP5KIβ expressing adenovirus. Recycling of IgA is not affected by PIP5KIβ. The graph shows the mean +/− SE of three experiments each performed in triplicate.
(TIF)

Figure S2 **ENTH and PTB domain binding to PtdIns(4,5)P_2_-containing and control liposomes.** Increasing concentrations of (A) ENTH-GST or (B) PTB-GST were incubated with control or PtdIns(4,5)P_2_-containing liposomes at room temperature for 30 min. The liposomes were pelleted and aliquots of the supernatant and pellet (2.6% and 20% of total, respectively) were separated by SDS-PAGE and stained with Coomassie blue. ENTH and PTB both bind to PtdIns(4,5)P_2_ containing liposomes in the pellet (P) fraction. ENTH and PTB are found in the pellet (P) and supernatant (S) fractions in control liposomes that do not contain PtdIns(4,5)P_2_.
(TIF)

Table S1 Quantitation of clathrin coated structure distribution in MDCK cells. MDCK cells cultured on permeable supports were infected with control or PIP5KIβ adenovirus and processed for electron microscopy. Clathrin coated structures were classified as shallow (type I), invaginated (type II), deeply invaginated (type III), or internalized and within one micron of the plasma membrane (type IV). Listed are the total number of clathrin coated structures counted of each type and the total length of membrane analyzed.
(DOC)
