# Supplementary figures and images for: PIP5KIβ Selectively Modulates Apical Endocytosis in Polarized Renal Epithelial Cells
Source: PLoS One. 2013 Jan 16;8(1):e53790. doi: 10.1371/journal.pone.0053790 (PMC3547069; doi:10.1371/journal.pone.0053790)

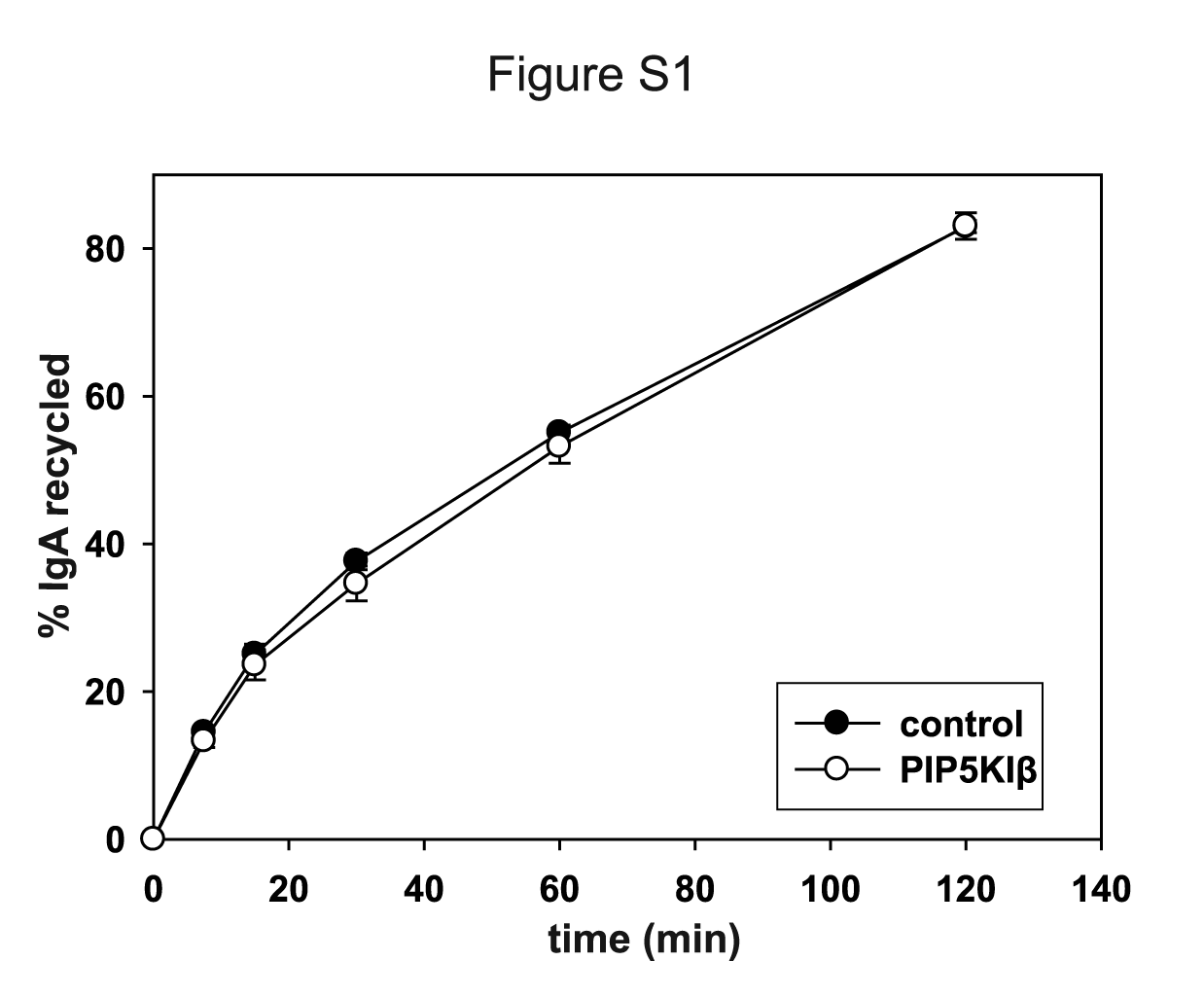

Supplement: Figure S1 — IgA Recycling is unaffected by PIP5KIβ. Recycling of apically-internalized 125I-IgA was quantified in polarized mCCD cells infected with adenovirus encoding the polymeric immunoglobulin receptor and either control or PIP5KIβ expressing adenovirus. Recycling of IgA is not affected by PIP5KIβ. The graph shows the mean +/− SE of three experiments each performed in triplicate. (TIF) [file pone.0053790.s001.tif]

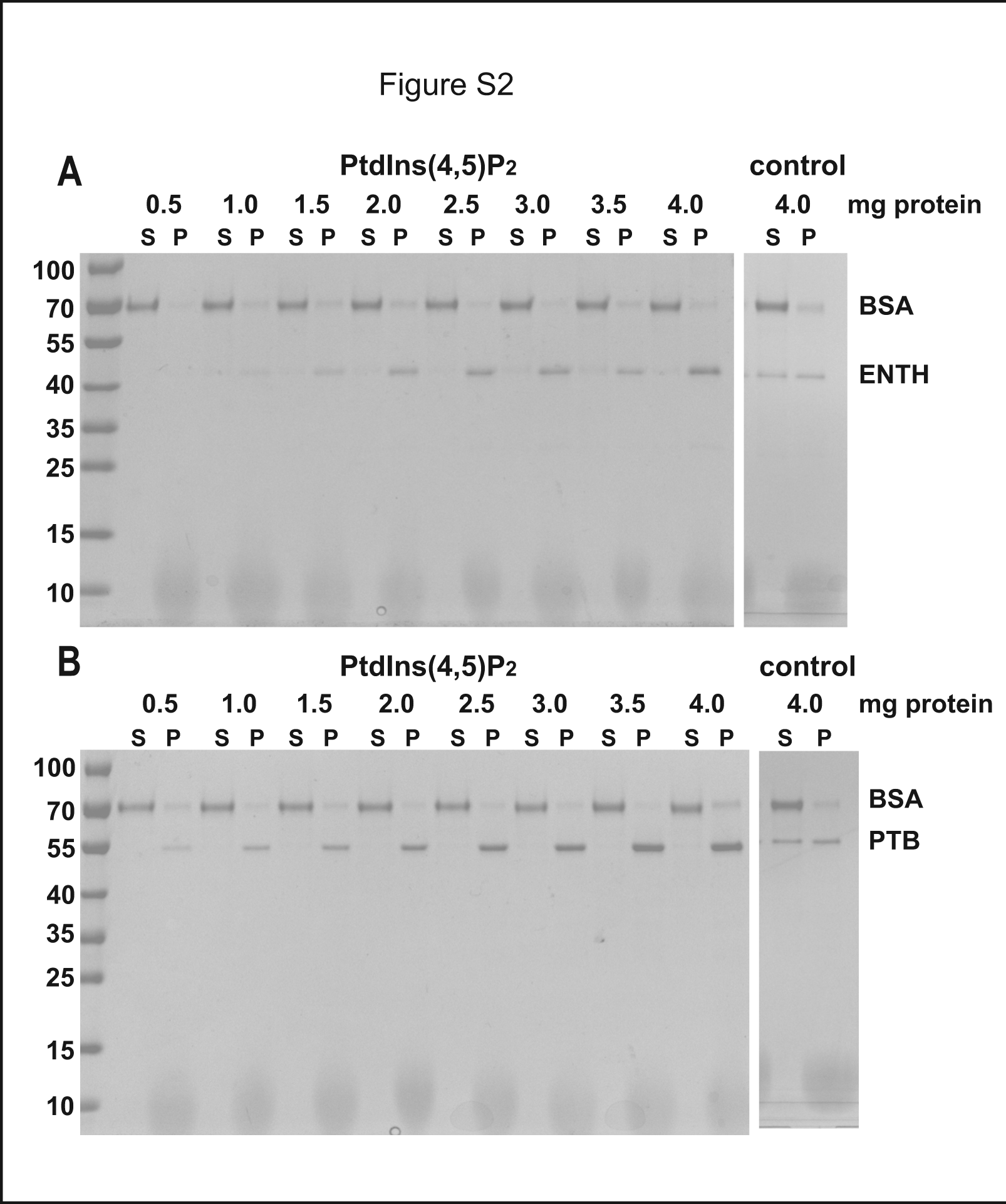

Supplement: Figure S2 — ENTH and PTB domain binding to PtdIns(4,5)P2-containing and control liposomes. Increasing concentrations of (A) ENTH-GST or (B) PTB-GST were incubated with control or PtdIns(4,5)P2-containing liposomes at room temperature for 30 min. The liposomes were pelleted and aliquots of the supernatant and pellet (2.6% and 20% of total, respectively) were separated by SDS-PAGE and stained with Coomassie blue. ENTH and PTB both bind to PtdIns(4,5)P2 containing liposomes in the pellet (P) fraction. ENTH and PTB are found in the pellet (P) and supernatant (S) fractions in control liposomes that do not contain PtdIns(4,5)P2. (TIF) [file pone.0053790.s002.tif]
