# Supplementary material for: PIP5KIβ Selectively Modulates Apical Endocytosis in Polarized Renal Epithelial Cells
Source: PLoS One. 2013 Jan 16;8(1):e53790. doi: 10.1371/journal.pone.0053790 (PMC3547069; doi:10.1371/journal.pone.0053790)
Supplement: Table S1 — Quantitation of clathrin coated structure distribution in MDCK cells. MDCK cells cultured on permeable supports were infected with control or PIP5KIβ adenovirus and processed for electron microscopy. Clathrin coated structures were classified as shallow (type I), invaginated (type II), deeply invaginated (type III), or internalized and within one micron of the plasma membrane (type IV). Listed are the total number of clathrin coated structures counted of each type and the total length of membrane analyzed. (DOC) [file pone.0053790.s003.doc]

**Table S1.**

|  | **Apical** | | **Basolateral** | |
| --- | --- | --- | --- | --- |
|  | **Control** | **PIP5KIβ** | **Control** | **PIP5KIβ** |
| **Class I** | 2 | 3 | 1 | 0 |
| **Class II** | 5 | 5 | 2 | 9 |
| **Class III** | 9 | 2 | 6 | 5 |
| **Class IV** | 26 | 22 | 20 | 32 |
| **Total** | 42 | 32 | 29 | 46 |
| **Membrane length (µm)** | 1377 | 1585 | 1469 | 1408 |
